# Traumatic hemipelvectomy: an appeal for primary completion

**DOI:** 10.1007/s00402-025-05850-8

**Published:** 2025-05-02

**Authors:** Jan Lindahl, Minna Laitinen, Axel Gänsslen, Dietmar Krappinger, Juha Kiiski, Mario Staresinic

**Affiliations:** 1 Department of Orthopaedics and Traumatology, Helsinki University Hospital, Helsinki, Finland; 2 University of Helsinki, Helsinki, Finland; 3 Department of Trauma Surgery, Hannover Medical School, Hannover, Germany; 4 Department of Orthopaedics and Traumatology, Innsbruck Medical University, Innsbruck, Austria; 5 Department of Musculoskeletal Surgery and Diseases, Tampere University Hospital, Tampere, Finland; 6 Tampere University, Tampere, Finland; 7 Department of General and Sports Traumatology, University Hospital “Merkur” Zagreb, Zagreb, Croatia

**Keywords:** Traumatic hemipelvectomy, Management, Case report, Debridement

## Abstract

Traumatic hemipelvectomy (TH) is a rare and extremely severe injury of the pelvic area, which is often life-threatening and associated with a high mortality rate. Individual treatment is focused on the pelvic fracture and the type of accompanying injuries. The management of these severely injured patients places a considerable challenge on the resuscitation team. Patient management should be aggressive from the start. Current literature is focused predominantly on survivors, with only few case series providing possible treatment recommendations. Aggressive initial treatment is focused on standardized damage-control procedures during the prehospital, emergency room, and initial surgical phase to prevent exsanguination and contamination; a massive transfusion protocol should also be initiated immediately to address traumatic coagulopathy. Standard vascular treatment addresses the vascular injury. Colostomy is often recommended for adequate soft-tissue trauma management. Attempts at limb salvage often result in higher complications rates with non-functional limbs compared with completion of the TH. Thus, in cases of critical ischemia and identified relevant sacral nervous plexus injury during initial debridement in predominantly open injuries, primary completion of the hemipelvectomy is recommended. Level of Evidence: IV.

## Introduction

Traumatic hemipelvectomy (TH) is a rare and extremely severe injury of the pelvic area [21, 22], which is often life-threatening with a high mortality rate. With ongoing improvements in prehospital care, an increasing number of severely injured patients survive such an injury and are transferred from the scene to the hospital.

Theodor Billroth first reported a case of TH in 1889 [19]; this patient survived only 6 h post-injury. One of the largest summaries of predominantly survivors after TH was analysed by Bakota et al. [2].

There is no accepted definition of TH. An open soft-tissue injury is not required.

Lipkowitz et al. defined TH as a “unique type of an open pelvic fracture with wide separation of one innominate bone from the pubic symphysis and sacrum, extensive disruption of the soft-tissues in the inguinal area, avulsion of the external iliac vessels, and severe stretch injury or disruption of the femoral and sciatic nerves” [12]. The vascular injury was considered more relevant and is often more severe than the accompanying neurological injury.

Rieger et al. defined TH as “an extreme and near-lethal form of an open pelvic fracture” [16], while Pohlemann et al. defined TH as an “unstable ligamentous or osseous hemipelvic injury with rupture of the pelvic neurovascular bundle (open or closed integuments)” [14]. Cavadas et al. distinguished between complete or incomplete TH injuries, with incomplete injuries being more common than complete injuries [3]. This injury may be associated with closed soft-tissues [20].

Bakota et al. defined TH as a unilateral (rarely bilateral) pelvic ring-type C-injury with an at least III° pelvic vascular injury (external iliac artery, affecting limb perfusion) in combination with an at least partial lumbosacral plexus injury [2].

In recent years, several additional survivors were reported in the literature without defining a uniform treatment concept. Treatment is highly individual, and a tendency of primary completion of TH should be considered.

We present two further TH cases after primary completion of such a devasting injury.

## Case one

A 28-year-old previously healthy, non-smoking man presented to our service following a high-speed motorcycle accident. The patient collided with a motor vehicle stopped at a red light. During the crash, he was ejected over the car, but his right lower extremity remained partially entrapped beneath the motorcycle handlebar. He was transported by ambulance to the emergency department’s resuscitation room for initial assessment.

Upon arrival, the primary survey was interrupted due to hemodynamic instability and the patient was taken directly to the operating room without imaging. The patient was wearing a leather driving suit, which had helped stabilize his lower extremities despite the presence of large open wounds.

**Fig. 1:**
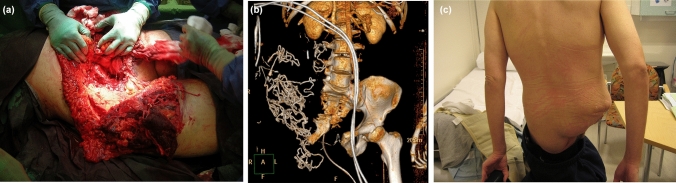
A 28-year-old male patient sustained a traumatic hemipelvectomy (TH) following a high-speed motorcycle accident. Due to haemodynamic instability, the patient was taken directly to the operating room without imaging. **a** Clinical photograph illustrating the extent of the primary large traumatic wound on the right hemipelvis with < 50% soft tissue connection between the trunk and lower extremity prior to amputation. Exploration revealed a complete disruption of the hemipelvis from the axial skeleton, discontinuity of the main blood flow by complete avulsion of femoral vessels, avulsion of the nerves, and ruptures of the colon and small intestine. **b** Computed tomography of the pelvis following hemipelvectomy revealed a small remnant of the right pubic bone and disruption of the contralateral sacroiliac joint. **c** Clinical photograph of the patient standing 1 year post-trauma

In the operating room, a large wound along the ilioinguinal line was observed (Fig. 1a). The fracture extended anteriorly through the symphysis pubis and posteriorly through the lateral sacrum (Fig. 1b). The rectum was exposed and both the colon and small intestine were ruptured, with three additional small lacerations identified in the small intestine. Nerve roots S1, L5, and L4 were torn at the foraminal level, while the ischial and femoral nerves were ruptured distally. Both the femoral artery and vein were transected.

An external hemipelvectomy was performed. The transected vessels were ligated, intestinal ruptures were sutured, and the skin was loosely closed. During the first surgery, a massive transfusion protocol was initiated, and the patient received 29 units of packed red blood cells, 10 units of frozen plasma, and 20 units of platelets. Initial laboratory results revealed severe metabolic acidosis (pH 7.18, base excess −16.8, lactate 7.3 mmol/L) (Table 1). The first haemoglobin measurement showed a haematocrit level of 0.33.

**Table 1:** Relevant laboratory parameters of case one during the initial 48 h course

Laboratory test	Arrive at OR	During surgery	2 h postop	4 h postop	1 st postop day	2nd postop day
Hemoglobin (g/L)	55	101	82	46	147	115
Platelet count (10^9^/L)	81	113	42	66	97	91
aPPT (s)		> 160		100		57
Fibrinogen (g/L)		0.4		< 0.4		1.7
Lactate (mmol/L)	6.8	7.0	7.2	4.7	1.2	1.2

*OR* operating room

Postoperative whole-body computed tomography (CT) identified additional injuries, including a small traumatic subarachnoid haemorrhage (SAH) and minor lacerations to the kidney and spleen, neither of which exhibited active bleeding and thus did not require further surgical intervention. A contralateral pelvic fracture was also noted (Fig. 1b). The course of relevant laboratory parameters during the first 48 h after admission is presented in Table 1.

The patient was treated in the intensive care unit (ICU) for 17 days and underwent eight additional surgeries. These included three bowel resections, three surgical wound revisions, and one osteosynthesis for metacarpal bone fractures. The patient developed bowel complications, including fistulae, which were managed conservatively.

He remained hospitalized for 6 months, followed by another 6 months in a rehabilitation unit. The patient was ultimately discharged home after 1 year (Fig. 1c). At 1.5 years post-trauma, four small intestinal fistulae were surgically treated successfully. However, he continued to experience persistent local skin issues, which were managed with topical treatments.

The patient was later diagnosed with ulcerative colitis and suffered from severe phantom pain, along with significant mental health challenges that hindered his rehabilitation progress. The patient died from causes unrelated to the initial injuries 7 years post-trauma.

## Case two

A 53-year-old previously healthy woman was struck by a bus after slipping and falling off the pavement under icy conditions. Upon arrival at the emergency department, her pelvis was clinically unstable. A focused assessment with sonography for trauma (FAST) was performed, revealing no bleeding in the abdominal or thoracic cavities. However, within minutes, the patient became hemodynamically unstable, with a heart rate of 124 beats per minute and a blood pressure of –/50 mmHg. She was immediately transferred to the operating room without further imaging. Initial laboratory tests indicated severe acidosis (pH 7.04, base excess − 20.0, lactate 11.3 mmol/L).

In the operating room, a large open wound along the ilioinguinal line was identified, accompanied by brisk bleeding from the iliac communis vessels. The wound extended into the lower abdomen. The bleeding vessels were proximally clamped, and a clinical assessment was conducted. The fracture line traversed the sacroiliac joint; all plexus roots and the femoral nerve were torn. Anteriorly, the fracture extended through the pubic and ischial bones. An external hemipelvectomy was performed, with the transected vessels secured. Intra-abdominal organs were intact, and a prophylactic loop transverse colostomy was created.

A massive transfusion protocol was initiated during surgery. Despite this protocol, the patient remained coagulopathic, necessitating pelvic packing. The abdominal wound was temporarily closed with a Bogota bag. The patient received 30 units of packed red blood cells, 17 units of frozen plasma, 24 units of platelets, recombinant activated Factor VII, and Factor XIII concentrate. Following these interventions, the patient’s haemodynamic status stabilized. Postoperatively, the patient was admitted to the ICU, where hemodynamic stability was achieved. However, she developed acute renal insufficiency requiring haemodialysis.

The course of relevant laboratory parameters during the first 48 h after admission is presented in Table 2.

**Table 2:** Relevant laboratory parameters of case two during the initial 48 h course

Laboratory test	Arrive at OR	During surgery	2 h postop	4 h postop	1 st postop day	2nd postop day
Hemoglobin (g/L)	38	97	79	35	88	64
Platelet count (10^9^/L)	72	142	89	29	63	93
aPPT (s)	61	67	105	69	48	38
Fibrinogen (g/L)	0.8	0.9	< 0.6	1.3	1.5	2.4
Lactate (mmol/L)	6.4	11.1	11.6	11.3	3.9	3.3

*OR* operating room

On the following day, a whole-body CT scan revealed a small subarachnoid haemorrhage (SAH) and a minor brain contusion, both of which were managed conservatively. On the second post-trauma day, a secondary assessment of the pelvic wound was conducted in the operating theatre. Upon swab removal, no active bleeding was observed, and all tissues, including the bowel, were viable. New pelvic packing was applied.

Pelvic packing was replaced on the third post-trauma day. During this procedure, the patient suffered a massive pulmonary embolism, leading to a sudden drop in oxygen saturation and increased carbon dioxide retention. She became haemodynamically unstable. A subsequent CT scan revealed an extensive thrombus completely occluding the right pulmonary vein.

Due to critical oxidative dysfunction, the patient was treated with an intravenous infusion of dalteparin to manage the coagulation cascade. Despite these interventions, she developed disseminated intravascular coagulation (DIC), resulting in further haemodynamic instability. The patient succumbed to her condition 7 days post-trauma.

## Review of case reports

Detailed epidemiological data of TH are missing, as most (case) reports are focused only on survivors of TH [17, 18, 21, 22]. There are few larger case series; such series allow conclusions on mortality.

Bakota et al. performed an extensive analysis based on 103 published cases with TH, which were mostly single cases of surviving patients. The following results were reported [2]:

- The main injury mechanisms include lower extremity forced hyperabduction injury combined with external rotation (e.g. unrestrained cyclist or motorcyclist), traction-type extremity avulsion from the fixed pelvis and crush (roll-over) mechanism; the predominant injury mechanisms were motorcycle injuries and injuries in patients without adequate protection (pedestrians, roll-over mechanisms).
- 4 of 5 patients were male
- The type of HP was complete in 31.2%, near total in 22.6%, and incomplete in 46.2%
- All patients presented with Tile type C injuries often with a C1.2 c5 injury (hemipelvic separation with complete SI joint + symphyseal disruption)
- Nearly every patient was in severe traumatic-haemorrhagic shock (grade III-IV according to ATLS)
- The average Injury Severity Score (ISS), if recorded, was 46.5 points, indicating a severe polytrauma situation with a high risk of mortality and coagulopathy
-  > 94% had pelvic vascular disruption (18 common iliac vessel injury, 10 internal iliac vessel injury, 57 external iliac vessel injury), 13 femoral vessel transection)
- 61% had documented lumbosacral plexus or peripheral pelvic nerve transsections
- Approximately 85% will have concomitant injuries to intrapelvic organs or soft tissues
- Approximately 30% will have concomitant intra-abdominal injuries
- Relevant additional injuries (e.g. head, chest extremities) were observed in over half of the patients
- If reported, a mean of 23.7 PRBC and a mean of 14.5 FFP were transfused during the initial perioperative treatment phase
- No standardized initial and secondary treatment concept could be derived
  - resuscitation according to standardized shock-room protocols (e.g. ATLS protocol)
  - classical damage control treatment with focus on bleeding and contamination control
  - initiation of a massive transfusion protocol
  - initial wound debridement
  - standard vascular treatment: vessel ligation and rarely vascular reconstruction
  - generous indication for colostomy (performed in approximately 80%) for adequate faecal diversion
  - primary pelvic ring reconstruction resulted in 89% secondary hemipelvectomy

The expected mortality rate is > 50% [10, 14, 24, 25]; only one recent study had a mortality rate < 20%, which was probably due to better treatment experience in polytrauma patients [7].

Several additional survivors of a TH were published during the last 5 years. The TH was initially treated by completion of the injury.

Smith described a 21-year-old woman after MVA presenting awake but in relevant shock with an open right anterior to posterior gluteal pelvic wound additionally involving the perineum [18]. The right limb was pulseless, even with Doppler ultrasound and complete sensoric and motoric deficits. A pelvic anterior–posterior X-ray revealed a complete right hemipelvic dissociation. Immediate entireesurgical exploration revealed an intact common iliac artery, disruption of most internal iliac artery branches, a disrupted common iliac vein, a thrombosed external iliac artery, transected and thrombosed external iliac and femoral veins, an intact femoral artery, a transsected sciatic and femoral nerve, complete bladder and urethral laceration, complete transection of the rectum, and vaginal wall laceration. Acute completion of the hemipelvectomy was performed with wound closure using a gluteal myocutaneous flap. Several wound revisions were necessary. Ultimate wound healing was reported after 3 months [18].

Ghimire et al. presented a 19-year-old female in severe shock after a crush injury with primary complete left hemipelvectomy transferred after 3 h to definitive care [6]. No attempt was made to reconstruct the mangled extremity.

Li et al. described a 4-year-old girl with a left partial (soft-tissue attachment) TH, which was secondary completed [11]. Compensatory scoliosis developed within 6 months.

Al-Wageeh et al. published a rare case of a near total traumatic left hemipelvectomy and a right-sided internal hemipelvectomy [1]. After initial completion of the left hemipelvectomy, a secondary right hemipelvectomy was performed after 24 h due to absent blood flow and bony instability. The patient survived and soft-tissue reconstruction was successfully performed using an anterolateral thigh flap.

Zheng et al. reported a 55-year-old male after a crush injury with complete right leg and hemipelvis avulsion in severe haemorrhagic shock at admission (ATLS grade IV) [26]. Emergency and surgical evaluation revealed a large dirty open wound starting from the ribs to the perineum with intact peritoneum. Active bleeding was observed and transection of the common iliac vessels with thrombosis of the internal iliac and external vessels was present. These vessels were ligated. Extensive debridement was performed and revealed an avulsed scrotum with exposed testis and sacrum after a C1.2c5 injury (complete hemipelvis dislocation). The wound was left open and several debridements were necessary due to infection and necrosis until wound coverage was possible using a split-skin graft from the patient’s left thigh. A diverting colostomy was placed on day 14. An external prosthetic replacement was used after discharge on day 72 allowing walking. Twelve months after the injury, he was able to perform daily activities and reported occasional phantom pain but no depressive signs.

Wan et al. reported a comparable injury after a motorcycle accident in 22-year-old female with an open complete right hemipelvis dislocation with transection of the external iliac vessels and sciatic and femoral nerve transection [23]. A loop transverse colostomy was placed on the left side of the abdomen, which was changed to a definitive permanent colostomy due to anal sphincter dysfunction. The further course was complicated due to local infection and skin necrosis, necessitating multiple re-debridements over 9 months until definitive wound closure was performed using a local flap. A rectovaginal cutaneous fistula was treated with fistulectomy and debridement. A right modular hemipelvis prosthesis was used for rehabilitation, which permitted even rock-wall climbing after 3 years.

In contrast to the patients described, Jansen et al. reported a successful case of limb salvage in a 14-month-old male toddler after TH [9]. After immediate bleeding control (clamping of external iliac vessels, aortic clamping), a child-adapted pelvic ring osteosynthesis was performed, followed by reconstruction of the vessel injury, reconstruction of the urethra, and reduction of both testicles. At 20 months, the leg was plegic and signs of femoral head necrosis were seen.

Herold et al. reported two survivors of TH after limb salvage [8]. After 1 year, both patients presented with a non-functional limb with severe senso-motoric complaints. It was concluded that “the question of limb salvage still remains an individual decision, requiring enormous experience both in the treatment of pelvic injuries as well as polytraumatized patients”.

Pfalzgraf et al. recently published a case after entrapment injury in a 34-year-old male patient sustaining a complex III° open left TH with a complete anterior hemipelvic dislocation and symphyseal disruption in association with ipsilateral avulsion of the iliac external vessels presenting with complete ischemia of the left leg and a right central sacrum fracture with suspected dissection of the iliac external artery [13]. After immediate left wound packing and balloon occlusion of the right common iliac artery, posterior fixation was performed using the pelvic C-clamp in combination with symphyseal plating. Intraoperative evaluation revealed complete sacral plexus dissection. Vascular reconstruction included a left arterial stent grafting and ligation of the external iliac vein. Additionally, a prophylactic bilateral lower leg fasciotomy was performed, followed by thigh fasciotomy. Due to soft-tissue trauma and reperfusion syndrome, multiple organ failure developed on the first postoperative day, primary with acute renal, liver, and cardiovascular failure. A few days later a left phlegmasia coerulea dolens resulted in completion of the left hemipelvectomy. The further course was complicated by open-wound treatment due to sacral osteomyelitis, fistula excision, and plastic reconstruction over 109 weeks.

The discussion of limb salvage versus amputation remains controversial. Galland et al. compared patients after limb salvage with 3 patients after completion of sustained TH [5]. Better general health, quality of life, and pelvic situation were observed after completion of TH.

Overall, analyses of case reports show that in cases of critical ischemia and identified relevant sacral plexus injury, primary completion of the hemipelvectomy is more often recommended under the concept “life before limb” [13].

## Review of case series

There are only four studies on consecutive cases of TH that were analysed from a whole group of pelvic-ring injury patients [7, 10, 14, 24]. Pohlemann et al. analysed 2002 patients with pelvic-ring injuries treated between 1972 and 1994. In this group, 11 patients suffered traumatic hemipelvectomies (0.5%) [14]. Labler et al. reported a higher incidence of 2.4% from a group of 373 pelvic-ring injuries treated between 1998 and 2004, of which 9 fulfilled the criteria of TH [10]. Wang et al. analysed 917 patients with pelvic-ring fractures treated between 2000 and 2011 and identified 9 cases (1%) with a TH (2 complete, 7 partial) [24]. Recently, He et al. reported 21 patients with a partial TH that was defined as a complete disruption of the hemipelvis from the axial skeleton caused by trauma, discontinuity of the main blood flow by complete or partial avulsion or secondary thromboembolism, avulsion or severe stretching of the nerves, and > 50% connection between the lower extremity and trunk by soft tissue [7].

Combining these data leads to an overall incidence of 0.9% (29/3292 cases). Only He et al. did not present data on their entire population of pelvic patients.

The overall mortality rate was 46% (Pohlemann 63.6%, Labler 77.8%, Wang 55.5%, He 19%). Only He et al. observed a mortality rate well below 50%.

Pohlemann et al. described seven non-survivors, of which four died during the resuscitation phase [14]. Two patients died during the first week after admission (one patient after 5 days due to protracted shock and one patient due to sepsis). One patient died after 6 weeks due to multi-organ failure.

The immediate decision for completion of TH influenced later rehabilitation, as fewer complications were observed and the length of hospital stay and the risk of social disruption was reduced [14].

In the analysis of Labler et al., 4/9 patients died during the resuscitation phase within the first 4 h after admission [10]. A further three patients died during the first week due to septic complications. Overall, two patients had initial completion of TH, while two had completion within the first week after trauma. The two survivors had completion of TH, while all secondary procedures or pelvic c-clamp stabilization led to mortality [10].

Wang et al. reported seven patients, of which two immediately died during shock-room resuscitation [24]. Completion of TH was performed on the first day in three patients and on the fifth day in one patient (after an attempt of limb salvage). The latter patient died from sepsis on day 10. Another patient, secondarily transferred on day 6 after trauma, developed relevant infection leading to secondary completion of TH after an attempt of limb salvage.

Additionally, in contrast to the reported older experience [10, 14, 24], He et al. published a consecutive series of 21 patients treated with partial TH [7]. The mean age was 31.3 years (range 15–61 years). Fifteen patients were directly admitted to hospital, while the remaining six patients were transferred from other hospitals. The mean ISS was 49.8 points, and all patients were in severe haemorrhagic and hypovolemic shock. Eight patients achieved pelvic ring stabilization with an external fixator. All patients had injury to the iliac vessels, while 16 had lumbosacral plexus injury.

Overall, four patients died (19%). The mean hospital stay of the survivors was 101.6 days. Analysis of the 17 survivors revealed initial limb salvage in 8 patients (38.1%), 5 of these (62.5%) required secondary procedures (1 hemipelvectomy, 3 hip disarticulations, 1 supracondylar amputation). Primary hip disarticulation was performed in four survivors; two required secondary hemipelvectomy (50%). Overall, five patients had primary completion of the hemipelvectomy [7]. Of the four non-survivors, two died immediately during resuscitation, one died after primary hip disarticulation, and one died due to multiple organ failure after primary hip disarticulation, followed by secondary hemipelvectomy.

Overall, analyses of case reports show that the primary limb salvage was associated with a relevant number of septic complications that often require secondary hemipelvectomy.

## Combat-related TH

In contrast to non-combat-related TH, the injury mechanisms of combat-related TH clearly differs after military, terror environment, or high-energy explosive blast injuries. War-related blast TH often present with bilateral mangled lower extremities and at least one severely injured upper extremity. Interestingly, an analysis of consecutive blast-related TH showed a relevant lower mortality rate of only 15.4% [4]. These injuries more often present with minor active bleeding, due to vascular or arterial spasm and vessel retraction with subsequent thrombosis. Depending on the fracture type, a subtotal hemipelvectomy is favoured without primary wound closure. A relevant risk of heterotopic ossification formation was observed in the long term [15].

## Literature-derived treatment protocol

The main treatment protocol includes standard open-fracture treatment of the pelvis with an early multidisciplinary approach, early surgical intervention, definitive treatment, and psychotherapy [7].

Based on the extensive literature analysis of Bakota et al., the following treatment recommendations can be recommended [2]:

1. Damage control treatment for exsanguination, shock, and haemorrhage.
2. Completion of the hemipelvectomy.
3. Damage control treatment for protection of infection (debridement).
4. Damage control treatment for protection of infection (colostomy).
5. Adequate wound care (primary/secondary closure).
6. Low-level planning of re-debridement.

## Conclusion

TH is still associated with a high mortality rate. Literature reports are focused predominantly on survivors and only a few case series provide possible treatment recommendations.

Attempts at limb salvage often result in high complication rates with non-functional limbs. Thus, in cases of critical ischemia and identified relevant sacral plexus injury during initial debridement in predominantly open injuries, primary completion of the hemipelvectomy is recommended.

## Data Availability

No datasets were generated or analysed during the current study.
